# Association of Night-Time Heart Rate With Ventricular Tachyarrhythmias, Appropriate and Inappropriate Implantable Cardioverter-Defibrillator Shocks

**DOI:** 10.3389/fcvm.2021.739889

**Published:** 2021-09-06

**Authors:** Xuerong Sun, Bin Zhou, Keping Chen, Wei Hua, Yangang Su, Wei Xu, Fang Wang, Xiaohan Fan, Hongxia Niu, Yan Dai, Zhimin Liu, Shuang Zhao, Shu Zhang

**Affiliations:** ^1^State Key Laboratory of Cardiovascular Disease, Arrhythmia Center, National Center for Cardiovascular Diseases, Fuwai Hospital, Chinese Academy of Medical Sciences and Peking Union Medical College, Beijing, China; ^2^Laboratory of Heart Center, Department of Cardiology, Zhujiang Hospital, Southern Medical University, Guangzhou, China; ^3^Department of Cardiology, Shanghai Institute of Cardiovascular Diseases, Zhongshan Hospital, Fudan University, Shanghai, China; ^4^Department of Cardiology, Nanjing Drum Tower Hospital, Nanjing, China; ^5^Department of Cardiology, Shanghai First People's Hospital, Shanghai Jiao Tong University School of Medicine, Shanghai, China

**Keywords:** night-time heart rate, implantable cardioverter-defibrillator, ventricular tachyarrhythmia, cardioverter-defibrillator therapy, cardiac autonomic activity

## Abstract

**Background:** Night-time heart rate (HR) is expected to reflect more accurately the cardiac autonomic function of modulating cardiovascular activity. Few studies have been conducted on the predictive values of night-time HR in relation to cardioverter-defibrillator therapies.

**Aims:** To explore the associations of night-time HR with the ventricular tachyarrhythmias (VTAs), appropriate and inappropriate implantable cardioverter-defibrillator (ICD) shocks.

**Methods:** Patients from the SUMMIT registry receiving ICD or cardiac resynchronization therapy with defibrillator (CRT-D) implantation were retrospectively analyzed using archived home monitoring data. Night-time HR was recorded from 2:00 am to 6:00 am during the first 30 to 60 days after implantation. VTA events and ICD shocks were identified using the intracardiac electrograms by two independent physicians. Restricted cubic splines and smooth curve fitting were conducted to address the non-linear associations between night-time HR and adjusted hazards for clinical outcomes.

**Results:** Over a mean follow-up duration of 55.8 ± 22.7 months, 187 deaths were observed among 730 patients. VTAs, appropriate and inappropriate ICD shocks were observed in 422 (57.8%), 293 (40.1%), and 72 (10.0%) patients, respectively. Apparent U-shaped non-linear associations of night-time HR with VTAs (P for non-linearity = 0.007), appropriate ICD shocks (P for non-linearity = 0.003) and inappropriate ICD shocks (P for non-linearity = 0.014) were detected. When night-time HR was beyond 60 bpm, every 1 bpm increase in night-time HR could result in 3.2, 3.3, and 4.9% higher risks of VTAs and appropriate and inappropriate ICD shocks, respectively; when night-time HR was lower than 60 bpm, every 1 bpm increase in night-time HR could result in 6.0 and 10.7% lower risks of appropriate and inappropriate ICD shocks. Compared to night-time HR of ≤ 50 or ≥70 bpm, night-time HR of 50–70 bpm was associated with 24.9, 30.2, 63.5, and 31.5% reduced incidences of VTA events, appropriate ICD shocks, inappropriate ICD shocks, and all-cause mortality, respectively.

**Conclusion:** Apparent non-linear associations of night-time HR with VTAs and ICD shocks were detected. An increasing incidence of VTAs and ICD shocks was observed at both low and high levels of night-time HR. Night-time HR of 50–70 bpm might be the optimal therapeutics target for the management of ICD/CRT-D recipients.

## Introduction

Life-threatening ventricular tachyarrhythmia (VTA) can be terminated by cardioverter-defibrillator therapies for patients with implantable cardioverter-defibrillator (ICD) implantation (1, 2). Cardioverter-defibrillator therapies have shown survival benefits in the prevention of sudden cardiac death (SCD), but ICD shocks and anti-tachycardia pacing (ATP) therapy remained associated with markedly increased risks of long-term mortality (1, 3–6). The SCD-HeFT study reported that after receiving an appropriate and inappropriate shock, ICD patients had a 5- and 2-fold higher risks of all-cause mortality, respectively (5). Kleemann et al. also observed that the occurrence of ATP therapy was associated with a 2.6 times higher mortality rate (4). Thus, the identification and management of the patients requiring cardioverter-defibrillator therapies are important to improve the prognosis after ICD implantation.

The heart rate (HR) is an individual physical sign as well as a non-invasive and affordable tool that can be easily measured (7). Some epidemiological studies have reported that high resting HR was associated with poor prognosis in cardiovascular events and all-cause mortality in subjects with or without cardiovascular diseases (8–10). Compared with resting HR and 24-h HR, night-time HR is measured during sleep periods and less likely to affected by diet, environmental factors, and physical and mental activities. Night-time HR is expected to reflect more accurately thee cardiac autonomic function of modulating cardiovascular activity (11–13). Furthermore, night-time HR rather than HR measured at other times proved to be a better prognostic mark for accessing cardiovascular risks in Johansen et al. and Palatini et al.'s studies (11, 12). However, few studies have focused on the predictive values of night-time HR in relation to cardioverter-defibrillator therapies.

In this study, the patients who received ICD or cardiac resynchronization therapy with defibrillator (CRT-D) for the primary or secondary prevention of SCD were analyzed. The night-time HR (02:00–06:00), VTAs and ICD therapy events were obtained from home monitoring recordings, aiming to explore the associations of night-time HR with VTAs requiring cardioverter-defibrillator therapies, appropriate/inappropriate ICD shocks, and all-cause mortality after ICD/CRT-D implantation.

## Methods

### Study Design

The Study of Home Monitoring System Safety and Efficacy in Cardiac Implantable Electronic Device-implanted Patients (SUMMIT) registry (Registration No. ChiCTR-ONRC-13003695) is an observational, prospective, and multicenter trial. Patients from the SUMMIT registry were retrospectively analyzed using the archived home monitoring data. The present study adhered to the principles of the Declaration of Helsinki, and it was approved by the ethics committees of Fuwai Hospital (the chief institute) and all other participating organizations. All patients provided written informed consent prior to enrollment.

### Study Participants

Patients who received ICD or CRT-D (Biotronik, Germany) implantation between May 2010 and May 2014 were included. ICD/CRT-D devices were implanted according to the guidelines' recommendations and equipped with a continuous home monitoring system. The percentage of average daily ventricular pacing in a single-chamber ICD was <10% or the percentage of average daily atrial and ventricular pacing percentage in a dual-chamber ICD was both <10% during the window period. Patients were excluded if they were <18 years old at implantation, got lost to follow-up, survived for <3 months, diagnosed with a malignant tumor, or scheduled for heart transplantation.

### Home Monitoring and Device Programming

After ICD/CRT-D implantation, a continuous home monitoring system was started immediately and transmitted information to the service center every day. Archived home monitoring data included HR, atrial and/or ventricular pacing, supraventricular episodes (atrial fibrillation [AF], atrial flutter [AFL], supraventricular tachycardia [SVT], and sinus tachycardia), VTAs, ATP therapy, ICD shock, etc. Routine follow-ups were also conducted via clinic visits or telephone interviews. If the transmission was interrupted, the research coordinator contacted the patients or family members to immediately confirm their health conditions.

The basic HR was 40–60 beats per minute (bpm). All patients received ventricular fibrillation (VF) and ventricular tachycardia (VT) monitor zones programmed independently of the device type. VT was detected at rates of ≥140 bpm, and VF was detected at rates of ≥200 bpm. In addition, ICD/CRT-D devices were equipped with the Biotronik SMART algorithm, which can distinguish VT/VF episodes from SVT episodes after analyzing the waveform and frequency of electrocardiograms (14).

### Night-Time and 24-Hour HR Measurement

Night-time HR was obtained from 2:00 am to 6:00 am when patients were at a sleep status. The 24-h HR was obtained from a 24-h period. Both mean values of night-time HR and 24-h HR were calculated as the average daily values during the first 30–60 days after ICD/CRT-D implantation.

### Grouping

Based on the cut-off values of night-time HR, acquired using restricted cubic splines and smooth curve fitting, patients were divided into three groups of different night-time HR levels: ≤ 50 bpm night-time HR group (*n* = 51), 50–70 bpm night-time HR group (*n* = 558), and ≥70 bpm night-time HR group (*n* = 121).

### Study Endpoints

The primary endpoints were the first VTA events and the first appropriate and inappropriate ICD shocks. The first VTA event was defined as the first identified VT/VF episode requiring cardioverter-defibrillator therapies (appropriate ATP therapy or ICD shock) after ICD/CRT-D implantation. Appropriate ICD shock was defined as the ICD shock delivered to the VTAs. Inappropriate ICD shock was defined as the ICD shock delivered for arrhythmias other than ventricular (sinus tachycardia, SVT, atrial tachycardia [AT], AF, AFL, etc.) or for non-arrhythmic events (noise, sensing problems, malfunction, etc.). The VTA events and appropriate and inappropriate ICD shocks were obtained from the intracardiac electrograms of tachycardia events, which were reviewed and further confirmed in a blinded manner by two cardiologists.

The secondary endpoint was all-cause mortality. The cause of death and the date of death were identified based on the death certificates supplied by family members.

### Statistical Methods

Continuous variables are presented as mean ± standard deviation, and categorical variables are presented as frequencies and percentages. One-way analyses of variance were performed to assess the differences between the continuous variables, and the chi-squared tests were used for the categorical variables. The association between night-time HR and 24-h HR was assessed using Pearson's correlation coefficient.

Restricted cubic splines and smooth curve fitting were conducted to explore the non-linear associations between night-time HR and adjusted hazards for clinical outcomes. If a non-linear relationship was detected, the inflection point was adopted as a dichotomizing cutoff value, and a 2-piecewise Cox proportional hazards model on both sides of the inflection point was constructed for a secondary analysis to further describe their non-linear associations. Multivariate Cox proportional hazard models were adjusted for independent, considerable variables with a *P* ≤ 0.05 in the univariate analysis.

The corresponding values of night-time HR, with lower limits of 95% confidence intervals (CIs) and Ln HR = 0, were obtained from the smooth curving fitting. Based on the adopted cut-off values, the patients were divided into three different groups of night-time HR levels. Kaplan–Meier survival curves with log–rank tests and univariate/multivariate Cox regression models were constructed to evaluate the predictive values of different night-time HR levels for different clinical outcomes, to explore the appropriate therapeutic targets for the management of ICD/CRT-D recipients.

Hazard ratios and 95% CIs were calculated to determine the impact. Statistical significance was set aat *P* < 0.05, and all tests were two-sided. Statistical analyses were conducted using SPSS Statistics version 23.0 (IBM Corp., Armonk, USA) and R version 4.0.3 (Bunny–Wunnies Freak Out, The R Foundation for Statistical Computing, Vienna, Austria).

## Results

### Baseline Characteristics

In this retrospective cohort study, 730 out of 1,015 consecutive patients receiving ICD or CRT-D implantation for primary or secondary prevention of SCD were included. Patients were excluded due to incomplete or unavailable home monitoring data (*n* = 227), >10% of average daily atrial or ventricular pacing percentage (*n* = 50), age at implantation <18 years old (*n* = 3), survival period of <3 months after device implantation (*n* = 3), and lost to follow-up (*n* = 2).

The mean age at implantation was 60.4 ± 13.9 years old, and male was dominant in this cohort (74.8%). ICD implantation was indicated in 537 patients (73.6%), with a mean left ventricular ejection fraction (LVEF) of 42.8 ± 14.9% and a mean left ventricular end-diastolic diameter (LVEDD) of 58.7 ± 13.2 mm. A total of 392 patients (53.7%) were diagnosed with heart failure (HF) before implantation, and 296 cases (40.5%) had HF reduced ejection fraction (HFrEF). A total of 425 patients (58.2%) received the device implantation for ICD secondary prevention. Among these patients, 120 (28.2%) had documented VF and resuscitated SCD, 230 (54.1%) had a history of documented sustained VT, and 75 (17.6%) had a history of unexplained syncope and could be induced to VT or VF during the electrophysiological study.

Table 1 illustrates the difference in baseline characteristics between the different groups of night-time HR levels. Patients with higher night-time HR levels were older at implantation (*P* = 0.006), and more patients in the higher night-time HR groups were implanted with CRT-D implantation (*P* < 0.001), ICD primary prevention ICD indication (*P* < 0.001), NYHA class III-IV (*P* < 0.001), lower LVEF (*P* < 0.001), larger LVEDD (*P* = 0.001), more comorbidities including HF (*P* < 0.001), HFrEF (*P* < 0.001), ischemic cardiomyopathy (ICM) (*P* = 0.039), percutaneous coronary intervention (PCI) (*P* = 0.013), prior AF (*P* = 0.046), more use of loop-diuretics (*P* < 0.001), digoxin (*P* = 0.011), but less preimplant syncope (*P* = 0.016), and amiodarone use (*P* = 0.019).

**Table 1:** Baseline characteristics.

	**Total**	**≤50 bpm night-time**	**50–70 bpm night-time**	**≥70 bpm night-time**	***P-*value**
	**(*N* = 730)**	**HR group (*n* = 51)**	**HR group (*n* = 558)**	**HR group (*n* = 121)**	
**HR monitoring data**					
Nighttime HR (bpm)	61.3 ± 9.1	47.9 ± 2.4	59.1 ± 5.0	77.0 ± 6.1	–
24-h HR	70.9 ± 9.8	57.1 ± 4.9	69.1 ± 6.8	84.8 ± 8.2	–
**Demographic characteristics**					
Age at implantation (years)	60.4 ± 13.9	54.7 ± 14.9	60.6 ± 14.0	62.0 ± 12.7	0.006
Sex, male (*n*, %)	546 (74.8%)		182 (74.9%)	176 (72.1%)	0.412
BMI (Kg/m^2^)	23.6 ± 3.0	23.8 ± 2.6	23.6 ± 3.1	23.2 ± 2.9	0.258
ICD implantation (*n*, %)	537 (73.6%)	50 (98.0%)	426 (76.3%)	61 (50.4%)	<0.001
Primary prevention (*n*, %)	305 (41.8%)	12 (23.5%)	236 (42.3%)	57 (47.1%)	<0.001
**Echocardiographic characteristics**					
LVEF (%)	42.8 ± 14.9	50.9 ± 13.6	43.6 ± 14.9	35.8 ± 12.7	<0.001
LVEDD (mm)	58.7 ± 13.2	52.7 ± 9.7	58.7 ± 13.3	61.3 ± 13.4	0.001
**Comorbidities**					
HF (*n*, %)	392 (53.7%)	11 (21.6%)	288 (51.6.%)	93 (76.9%)	<0.001
HFrEF (*n*, %)	296 (40.5%)	7 (13.7)	206 (36.9%)	83 (68.6%)	<0.001
NYHA class III-IV (*n*, %)	362 (49.6%)	11 (21.6%)	264 (47.3%)	87 (71.9%)	<0.001
Hypertension (*n*, %)	228 (31.2%)	10 (19.6%)	178 (31.9%)	40 (33.1%)	0.173
DM (*n*, %)	76 (10.4%)	5 (9.8%)	57 (10.2%)	14 (11.6%)	0.897
Stroke (*n*, %)	16 (2.2%)	0 (0.0%)	16 (2.9%)	0 (0.0%)	0.080
DCM (*n*, %)	171 (23.4%)	9 (17.6%)	129 (23.1%)	33 (27.3%)	0.372
HCM (*n*, %)	29 (4.0%)	4 (7.8%)	24 (4.3%)	1 (0.8%)	0.071
ICM (*n*, %)	247 (33.8%)	9 (17.6%)	197 (35.3%)	41 (33.9%)	0.039
Prior MI (*n*, %)	102 (14.0%)	3 (5.9%)	79 (14.2%)	20 (16.5%)	0.178
PCI (*n*, %)	65 (8.9%)	1 (2.0%)	46 (8.2%)	18 (14.9%)	0.013
CABG (*n*, %)	8 (1.1%)	0 (0.0%)	7 (1.3%)	1 (0.8%)	0.679
Valve disease (*n*, %)	16 (2.2%)	0 (0.0%)	13 (2.3%)	3 (2.5%)	0.538
Prior AF (n, %)	83 (11.4%)	3 (5.9%)	59 (10.6%)	21 (17.4%)	0.046
Preimplant presyncope (*n*, %)	41 (5.6%)	4 (7.8%)	32 (5.7%)	5 (4.1%)	0.608
Preimplant syncope (*n*, %)	151 (20.7%)	17 (33.3%)	117 (21.0%)	17 (14.0%)	0.016
**Medication**					
Betablockers (*n*, %)	413 (56.6%)	32 (62.7%)	313 (56.1%)	68 (56.2%)	0.654
ACEI/ARBs (*n*, %)	257 (35.2%)	16 (31.4%)	189 (33.9%)	52 (43.0%)	0.138
Aldosterone antagonists (*n*, %)	260 (35.6%)	12 (23.5%)	198 (35.5%)	50 (41.3%)	0.083
CCBs (*n*, %)	61 (8.4%)	4 (7.8%)	44 (7.9%)	13 (10.7%)	0.583
Statins (*n*, %)	169 (23.2%)	11 (21.6%)	128 (22.9%)	30 (24.8%)	0.874
Loop-diuretics (*n*, %)	189 (25.9%)	9 (17.6%)	132 (23.7%)	48 (39.7%)	<0.001
Digoxin (*n*, %)	141 (19.3%)	7 (13.7%)	99 (17.7%)	35 (28.9%)	0.011
Amiodarone (*n*, %)	212 (29.0%)	23 (45.1%)	160 (28.7%)	29 (24.0%)	0.019
Antiplatelets (*n*, %)	152 (20.8%)	10 (19.6%)	116 (20.8%)	26 (21.5%)	0.962

*ACEIs, angiotensin-converting enzyme inhibitors; AF, atrial fibrillation; ARBs, angiotensin receptor blockers; BMI, body mass index; bpm, beats per minute; CABG, coronary artery bypass grafting; CCB, calcium channel blockers; CRT-D, cardiac resynchronization therapy defibrillator; DCM, dilated cardiomyopathy; DM, diabetes mellitus; HCM, hypertrophic cardiomyopathy; HF, heart failure; HFrEF, heart failure with reduced ejection fraction; HR, heart rate; ICM, ischemic cardiomyopathy; LVEDD, left ventricular end-diastolic dimension; LVEF, left ventricular ejection fraction; MI, myocardial infarction; PCI, percutaneous coronary intervention*.

### HR and Clinical Outcomes

The mean values of night-time HR and 24-h HR were 61.3 ± 9.1 and 70.9 ± 9.8 bpm, respectively. Night-time HR was closely correlated with 24-h HR, detected by Pearson correlation coefficient (Pearson's *r* = 0.868, *P* < 0.001).

During a mean follow-up period of 55.8 ± 22.7 months, the VTA events requiring appropriate cardioverter-defibrillator therapies were observed in 422 patients (57.8%). A total of 293 patients (40.1%) experienced appropriate ICD shocks, and 72 patients (10.0%) experienced inappropriate ICD shocks. Inappropriate ICD shocks were triggered by AF/AFL/AT/SVT with rapid ventricular conduction (*n* = 58), sinus tachycardia (*n* = 1), T-wave oversensing (*n* = 2), and interruption (*n* = 11). Regarding mortality, 187 deaths (25.6%) occurred among 730 ICD/CRT-D recipients. The most common cause of death was progressive HF (41.2%).

The incidences of VTAs, appropriate and inappropriate ICD shocks, and all-cause mortality were compared across the three groups of different night-time HR levels. Compared to those in ≤ 50 bpm or ≥70 bpm night-time HR groups, the incidences of VTA events (62.7 vs. 56.6 vs. 61.2%, *P* = 0.501) and appropriate ICD shocks (49.0 vs. 38.4 vs. 44.6%, *P* = 0.180) in the 50–70 bpm night-time HR group were lower but not significant. Patients in the 50–70 bpm night-time HR group had a significantly lower incidence of inappropriate ICD shocks (19.6 vs. 7.7 vs. 16.5%, *P* = 0.001). However, the incidence of death from any cause increased continuously from 15.7 to 24.0 to 37.2% in the ≤ 50, 50–70, and ≥70 bpm night-time HR groups, respectively (*P* = 0.003).

### Non-linear Association of Night-Time HR With Clinical Outcomes

**Figure 1:**
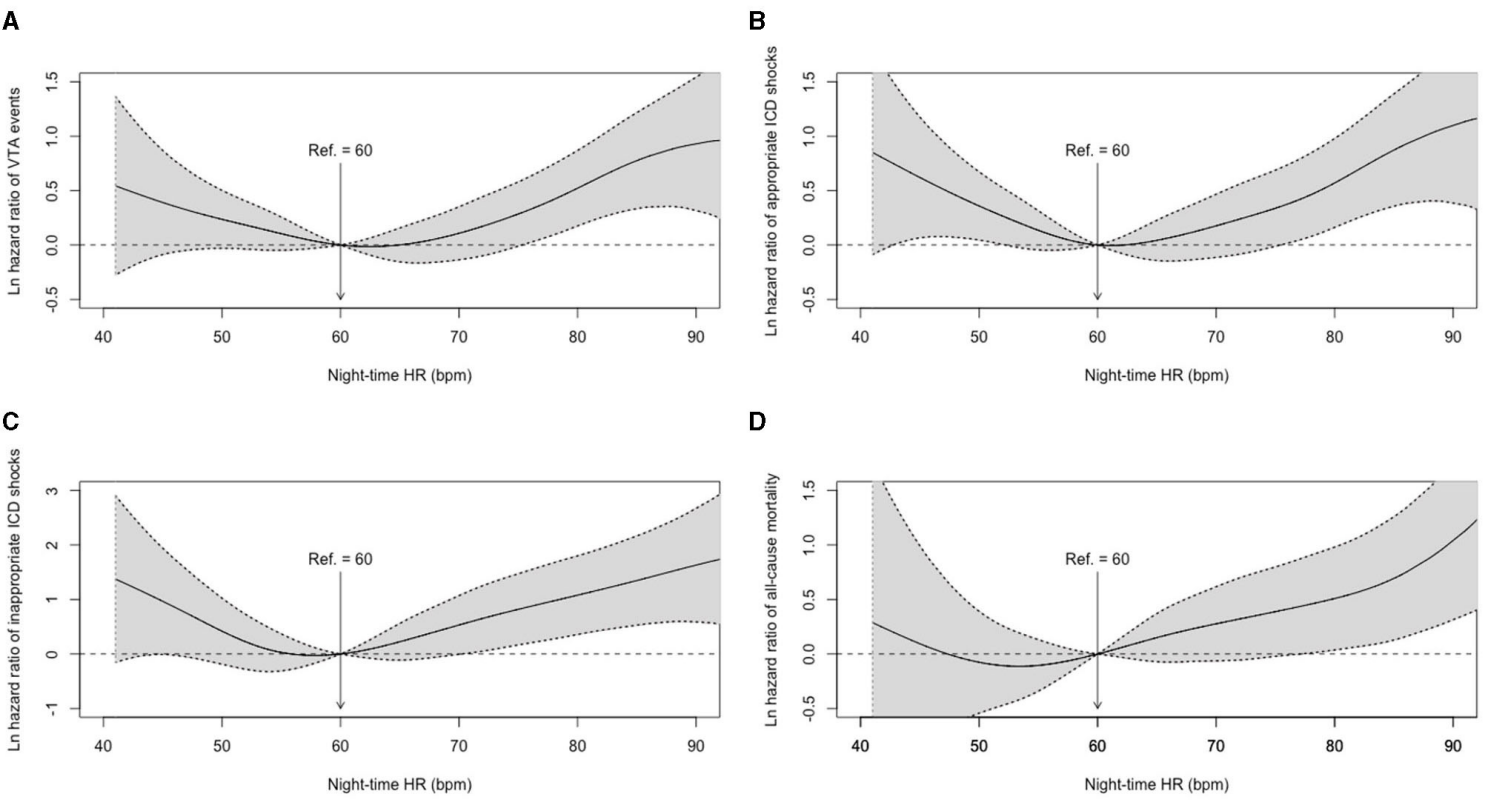
Adjusted hazard ratios for night-time HR in clinical outcomes. **(A)** Smooth curve fitting of VTA events; **(B)** Smooth curve fitting of appropriate ICD shocks; **(C)** Smooth curve fitting of inappropriate ICD shocks; **(D)** Smooth curve fitting of all-cause mortality. HR, heart rate; ICD, implantable cardioverter defibrillator; VTA, ventricular tachyarrhythmia.

Restricted cubic spline regression analysis was conducted to detect the non-linear associations between night-time HR and adjusted hazard ratios for VTA events, appropriate and inappropriate ICD shocks, and all-cause mortality. The results of the smooth curve fitting are shown in Figure 1. Apparent non-linear associations of night-time HR with the adjusted hazards for the first VTA events (P non-linearity = 0.007), the first appropriate ICD shock (P for non-linearity = 0.003), and the first inappropriate ICD shock (P for non-linearity = 0.014) were detected with a typical U-shaped curve when adjusted for valuable variables, including age at implantation, sex, LVEF, LVEF, prior AF and preimplant syncope. However, a non-linear relationship between night-time HR and all-cause mortality was not detected (P for non-linearity = 0.352).

Based on the U-shaped smooth curve fitting, the inflection points for VTA events, appropriate and inappropriate ICD shocks were detected at 62.0, 61.0, and 58.0 bpm of night-time HR, respectively. Therefore, 60 bpm was adopted as a dichotomizing cutoff value to further describe the non-linear associations in a secondary analysis (Table 2).

**Table 2:** Night-time HR's effects on VTAs and ICD shocks.

**Night-time HR** (per 1 bpm/increase)*	**Univariate Cox regression analysis**		**Multivariate Cox regression analysis**	
	Hazard ratio (95%CI)*	***P*-value**	Hazard ratio (95%CI)*	***P*-value**
**Night-time HR < 60 bpm (** ***n =*** **364)**				
VTA events	0.977 (0.944–1.010)	0.172	0.967 (0.933–1.001)	0.060
Appropriate ICD shock	0.960 (0.923–0.999)	0.045	0.940 (0.901–0.980)	0.004
Inappropriate ICD shock	0.901 (0.827–0.981)	0.017	0.893 (0.817–0.976)	0.013
**Night–time HR** ≥ **60 bpm (*****n =*** **366)**				
VTA events	1.034 (1.016–1.053)	<0.001	1.032 (1.013–1.052)	0.001
Appropriate ICD shock	1.035 (1.013–1.057)	0.002	1.033 (1.011–1.056)	0.004
Inappropriate ICD shock	1.049 (1.014–1.085)	0.006	1.049 (1.012–1.087)	0.009

*Multivariate Cox regression analysis was adjusted for age at implantation, sex, LVEF, LVEDD, prior AF, and preimplant syncope. AF, atrial fibrillation; CI, confidence interval; HR, heart rate; LVEDD, left ventricular end-diastolic dimension; LVEF, left ventricular ejection fraction; VTA, ventricular tachyarrhythmia*.

* *per 1 bpm increase in night-time HR*.

In patients with night-time HR < 60 bpm, night-time HR was shown to be a protective factor in appropriate ICD shock (hazard ratio = 0.960, 95% CI: 0.923–0.999, *P* = 0.045) and inappropriate ICD shock (hazard ratio = 0.901, 95% CI: 0.827–0.981, *P* = 0.017) in univariate Cox regression model. However, no significant difference was observed in VTA events (hazard ratio = 0.977, 95% CI: 0.944–1.010, *P* = 0.172). In multivariate Cox regression models, adjusted for age at implantation, sex, LVEF, LVEDD, prior AF, and preimplant syncope, the results remained consistent. Every 1 bpm increase in night-time HR could result in 6.0 and 10.7% decreased risks of appropriate ICD shock (hazard ratio = 0.940, 95% CI: 0.901–0.980, *P* = 0.004) and inappropriate ICD shock (hazard ratio = 0.893, 95% CI: 0.817–0.976, *P* = 0.013), respectively.

In patients with night-time HR ≥ 60 bpm, night-time HR was shown to be an independent risk factor for VTA events (hazard ratio = 1.034, 95% CI: 1.016–1.053, *P* < 0.001), appropriate ICD shock (hazard ratio = 1.035, 95% CI: 1.013–1.057, *P* = 0.002), and inappropriate ICD shock (hazard ratio = 1.049, 95% CI: 1.014–1.085, *P* = 0.006). After adjusting for considerable variables in the multivariate models, the results remained consistent. Each additional 1 bpm increase in night-time HR could result in 3.2, 3.3, and 4.9% higher risks of VTA events (hazard ratio = 1.032, 95% CI: 1.013–1.052, *P* = 0.001), appropriate ICD shocks (hazard ratio = 1.033, 95% CI: 1.011–1.056, *P* = 0.004), and inappropriate ICD shocks (hazard ratio = 1.049, 95% CI: 1.012–1.087, *P* = 0.009), respectively.

### Predictive Values of Night-Time HR of 50–70 bpm

The corresponding values of night-time HR were 50.0 and 75.3 bpm when the lower limits of 95% CI with Ln HR for the VTA events were zero; the cut-off values for appropriate ICD shocks were 52.2 and 75.4 bpm; and the cut-off values of inappropriate shocks were 45.0 and 70.2 bpm. After considering the obtained cut-off values comprehensively, night-time HR of 50–70 bpm was adopted to evaluate its predictive values in the following Kaplan-Meier survival analysis and Cox regression analysis.

**Figure 2:**
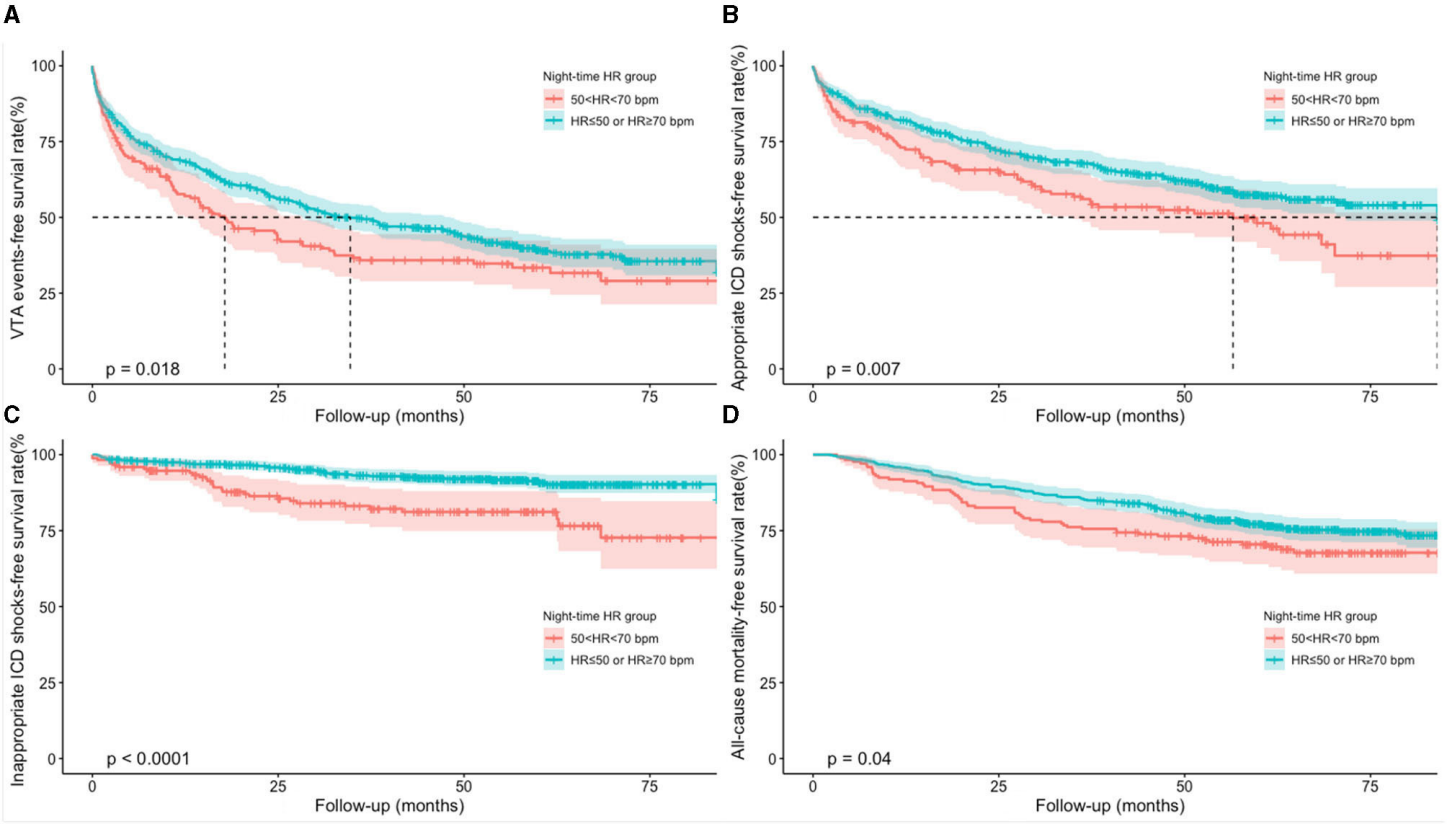
Accumulative incidences of clinical outcomes using Kaplan-Meier survival analysis. **(A)** Kaplan-Meier curves of VTA events; **(B)** Kaplan-Meier curves of appropriate ICD shocks; **(C)** Kaplan-Meier curves of inappropriate ICD shocks; **(D)** Kaplan-Meier curves of all-cause mortality. HR, heart rate; ICD, implantable cardioverter defibrillator; VTA, ventricular tachyarrhythmia.

The accumulative incidences of VTAs, appropriate and inappropriate ICD shocks, and death from any cause were compared using Kaplan–Meier survival analysis (Figure 2). Compared to those with ≤ 50 bpm or ≥70 bpm night-time HR, patients with 50–70 bpm night-time HR group had significantly lower accumulative incidence rates of VTA events (log rank, *P* = 0.018), appropriate ICD shocks (log rank, *P* = 0.007), inappropriate ICD shock (log rank, *P* < 0.001), and all-cause mortality (log rank, *P* = 0.040).

Univariate and multivariate Cox regression models were used to explore the predictive values of night-time HR of 50–70 bpm in clinical outcomes (Table 3). Univariate Cox regression analysis demonstrated that patients with 50–70 bpm night-time HR had significantly lower incidences in VTA events (hazard ratio = 0.768, 95% CI: 0.616–0.957, *P* = 0.019), appropriate ICD shocks (hazard ratio = 0.703, 95% CI: 0.542–0910, *P* = 0.007), inappropriate ICD shocks (hazard ratio = 0.370, 95% CI: 0.232–0.591, *P* < 0.001), and all-cause mortality (hazard ratio=0.718 95% CI: 0.522–0.987, *P* = 0.041), compared to those with night-time HR of ≤ 50 or ≥70 bpm. After adjusted for age at implantation, sex, ICD primary prevention, ICD indication, LVEF, LVEDD, diabetes mellitus (DM), ischemic cardiomyopathy (ICM), myocardial infarction (MI), prior AF, preimplant syncope, use of angiotensin-converting enzyme inhibitors or angiotensin receptor blockers (ACEI/ARBs), aldosterone antagonists, loop-diuretics, and digoxin, the results remained consistent. Night-time HR of 50–70 bpm was associated with 24.9, 30.2, 63.5, and 31.5% reduced incidences of VTA events, appropriate ICD shocks, inappropriate ICD shocks, and all-cause mortality, respectively, compared to those with night-time HR of ≤ 50 or ≥70 bpm.

**Table 3:** Predictive values of 50–70 bpm of night-time HR for clinical outcomes.

**Night-time HR** **(50–70 bp vs. ≤50 or ≥70 bpm)**	**Univariate Cox regression analysis**		**Multivariate Cox regression analysis**	
	**Hazard ratio (95%CI)**	***P*-value**	**Hazard ratio (95%CI)**	***P*-value**
VTA events	0.768 (0.616–0.957)	0.019	0.751 (0.600–0.941)	0.013
Appropriate ICD shock	0.703 (0.542–0.910)	0.007	0.698 (0.536–0.909)	0.008
Inappropriate ICD shock	0.370 (0.232–0.591)	<0.001	0.365 (0.225–0.592)	<0.001
All–cause mortality	0.718 (0.522–0.987)	0.041	0.685 (0.492–0.953)	0.025

*Multivariate Cox regression analysis was adjusted for age at implantation, sex, ICD indication, ICD primary prevention, LVEF, LVEDD, DM, ICM, MI, prior AF, preimplant syncope, usages of ACEI/ARBs, aldosterone antagonists, loop-diuretics, and digoxin. ACEIs, angiotensin-converting enzyme inhibitors; AF, atrial fibrillation; ARBs, angiotensin receptor blockers; CI, confidence interval; DM, diabetes mellitus; HR, heart rate; ICM, ischemic cardiomyopathy; LVEDD, left ventricular end-diastolic dimension; LVEF, left ventricular ejection fraction; MI, myocardial infarction; VTA, ventricular tachyarrhythmia*.

## Discussion

In this cohort, the associations of night-time HR with VTA events, appropriate and inappropriate ICD shocks, and all-cause mortality were explored using restricted cubic splines and smooth curve fitting in 730 ICD/CRT-D recipients. First, the apparent non-linear associations of night-time HR with VTAs, appropriate and inappropriate ICD shocks were detected with a typical U-shaped curve. Second, when night-time HR was beyond 60 bpm, every 1 bpm increase in night-time HR could significantly contribute to 3.2, 3.3, and 4.9% higher risks of VTA events, appropriate and inappropriate ICD shocks; and when night-time HR was below 60 bpm, every 1 bpm increase in night-time HR was associated with 6.0 and 10.7% reduced risks of appropriate and inappropriate ICD shocks, respectively. Third, night-time HR of 50–70 bpm could result in 24.9, 30.2, 63.5, and 31.5% lower incidences of VTA events, appropriate ICD shocks, inappropriate ICD shocks, and all-cause mortality, respectively, compared to those with night-time HR of ≤ 50 or ≥70 bpm.

In the present study, night-time HR was obtained in ICD/CRT-D patients. The home monitoring system was able to supply continuous recordings of HR, and night-time HR was acquired from 2:00 am to 6:00 am during sleeping periods. Night-time HR may not be influenced by diet, physical activity, or mental stress, etc. (11, 12). It could reflect the circadian rhythm of HR better than resting HR or 24-h HR (11, 12). However, compared to night-time HR, resting HR has been discussed more widely in previous studies. Resting HR could predict longevity and cardiovascular diseases in healthy individuals or patients with cardiovascular conditions (8–10). High resting HR has been associated with the development of different clinical events, including HF, hospitalizations for HF, MI, cardiovascular death, and all-cause mortality (7, 15–17). An analysis from ONTARGET and TRANSCEND demonstrated that whether in diabetic or non-diabetic individuals, the risks of cardiovascular death, MI, hospitalizations for HF and all-cause mortality rose when resting HR was above 75–80 bpm (16). The Melbourne Collaborative Cohort Study discovered that the individuals with temporal increases in resting HR over a decade had higher risks of death in the general population (17). In patients with HFrEF, the SHIFT trial showed that every 5-bpm increase in HR resulted in 16% higher risk of HF hospitalizations and cardiovascular death, and the risks was decreased after ivabradine treatment (18). The Swedish Heart Failure Registry reported that β-blocker use was associated with reduced mortality in HFrEF (19). Regarding VTA events, Beinart et al. observed that resting HR >63 bpm (prior to ICD/CRT-D implantation) could lead to 81 and 76% increased risks of VT/VF events and appropriate ICD therapy, respectively, compared to resting HR ≤ 63 bpm, but did not discuss appropriate and inappropriate ICD shock events (20). Most previous studies paid attention on the negative effects of elevated resting HR. Fewer studies obtained the HR value during night-time hours and evaluate its predictive values using restricted cubic splines and smooth curve fitting.

Patients with higher night-time HR were generally older and had poor physical conditions, more comorbidities and medication. Additionally, night-time HR showed a positive correlation with all-cause mortality, which was consistent with the previous findings (16, 17). The underlying mechanisms of negative effects might be that elevated resting HR could reflect or lead to progressed or accelerated oxidative stress, vascular stiffness, left ventricular dysfunction, endothelial dysfunction, systemic inflammation, plaque rupture, myocardial oxygen consumption, and cardiac autonomic dysfunction (7, 21). In contrast to previous studies, the associations of night-time HR with VTAs, appropriate and inappropriate ICD shocks were detected in ICD/CRT-D recipients using restricted cubic splines and smooth curve fitting. The apparent non-linear associations demonstrated an increased incidence of VTA events, appropriate and inappropriate ICD shock at both low and high levels of night-time HR, with risks rising progressively when night-time HR was beyond 60 bpm. The current evidence supports that high night-time HR could result in increased risks of VTAs requiring ICD therapies, which remained consistent in MADIT-RIT trial (20). High night-time HR in sinus rhythm were related to excessive sympathetic activation and depressed parasympathetic activity, probably contributing to the development of VTAs and subsequent appropriate ICD therapies (13, 21). Inappropriate ICD shocks might be triggered by atrial tachyarrhythmia with rapid ventricular conduction, which could be directly influenced by cardiac autonomic activity (5).

This study also discovered that the patients with low night-time HR (≤ 50 bpm) had increased incidences of VTAs, ICD shocks and mortality. Such typical J-shaped non-linear relationships between resting HR and cardiovascular outcomes were previously reported in HF patients (20, 21). A meta-analysis from three prospective cohorts (Cardiovascular Health Study, Health ABC study, and Kuopio Ischemic Heart Disease Study) (21) and an analysis from ONTARGET and TRANSCEND (16) demonstrated increased risks of cardiovascular outcomes at low levels of resting HR < 60 bpm. One possible explanation for this result might be that more patients with low resting HR were combined with sinus node or conduction diseases (21). Furthermore, the patients in the low night-time HR group (≤ 50 bpm) had more amiodarone use but less use of ACEI/ARBs, aldosterone antagonists, loop-diuretics, and digoxin, compared to the moderate and high night-time HR groups. Another possible mechanism might be attributed to the complex medication, which could influence the night-time HR through the autonomic nervous system.

ICD/CRT-D devices were able to supply continuous night-time HR monitoring data. The present study emphasized the importance of night-time HR monitoring after ICD/CRT-D implantation (17). Night-time HR could assist in identifying the individuals with increasing risks of VTAs, appropriate ICD shock, as well as inappropriate ICD shock, which was beneficial to improve the longevity. Regarding the optimal night-time HR management targets, the smooth curve fitting showed the lowest incidences of VTAs, ICD shocks and mortality when night-time HR was maintained at ~60 bpm. However, it was impractical to maintain the night-time HR at 60 bpm in real-world clinical applications. Then, the cut-off values of night-time HR were adopted based on the smooth curve fitting, and their predictive values were explored further. Multivariate Cox regression analysis showed that night-time HR of 50–70 bpm was associated with 25–64% reduced incidences of VTA events and ICD shocks. Appropriate night-time HR of 50–70 bpm might be the optimal therapeutic targets for the management of ICD/CRT-D recipients in clinical practice.

## Limitation

There are some limitations in this study. First, a prospective study is required to explore the therapeutic target values of the night-time HR after ICD/CRT-D implantation. Second, more studies are required to further explain the underlying mechanisms, especially for the patients with a relatively high incidence of VTAs, appropriate and inappropriate ICD shocks in the low night-time HR (≤ 50 bpm) group. Third, although the multivariate analyses were adjusted for potential confounding variables to minimize their imbalance, ICD indications were not balanced across the three groups. Caution should be exercised while generalizing the results to populations other than ICD/CRT-D recipients.

## Conclusion

Apparent non-linear associations of night-time HR with VTAs and ICD shocks were detected. Increasing incidences of VTAs, appropriate and inappropriate ICD shocks were observed at both low and high levels of night-time HR in ICD/CRT-D recipients. Night-time HR of 50–70 bpm might be the optimal therapeutics targets for the management of ICD/CRT-D recipients.

## Data Availability Statement

The original contributions presented in the study are included in the article/supplementary material, further inquiries can be directed to the corresponding author/s.

## Ethics Statement

The studies involving human participants were reviewed and approved by The present study, which conformed to the Declaration of Helsinki, was approved by the ethics committee of Fuwai Hospital (the chief institute) and all other participating organizations (Zhongshan Hospital Fudan University, Nanjing Drum Tower Hospital, Shanghai First People's Hospital et al.). The patients/participants provided their written informed consent to participate in this study. The patients/participants provided their written informed consent to participate in this study.

## Author Contributions

XS, BZ, and SZhao performed the conception or design of the work. XS, BZ, KC, WH, YS, WX, FW, XF, HN, YD, ZL, and SZhang contributed to the acquisition, analysis, and interpretation of data for the work. XS and BZ drafted the manuscript. SZhao and SZhang critically revised the manuscript. All authors gave final approval and agreed to be accountable for all aspects of work ensuring integrity and accuracy.

## Conflict of Interest

The authors declare that the research was conducted in the absence of any commercial or financial relationships that could be construed as a potential conflict of interest.

## Publisher's Note

All claims expressed in this article are solely those of the authors and do not necessarily represent those of their affiliated organizations, or those of the publisher, the editors and the reviewers. Any product that may be evaluated in this article, or claim that may be made by its manufacturer, is not guaranteed or endorsed by the publisher.

## Abbreviations

ACEI/ARB: angiotensin-converting enzyme inhibitors or angiotensin receptor blockers
AF: atrial fibrillation
ATP: antitachycardia therapy
BMI: body mass index
CABG: coronary artery bypass grafting
CCBs: calcium channel blockers
CI: confidence interval
HR: heart rate
CRT-D: cardiac resynchronization therapy defibrillators
DCM: hypertrophic cardiomyopathy
DM: diabetes mellitus
HCM: hypertrophic cardiomyopathy
HF: heart failure
HFrEF: heart failure with reduced ejection fraction
ICD: implantable cardioverter-defibrillator
ICM: ischaemic cardiomyopathy
LVEDD: left ventricular end diastolic diameter
LVEF: left ventricular ejection fraction
MI: myocardial infarction
NYHA class: New York Heart Association class
PCI: percutaneous coronary intervention
VTA: ventricular tachyarrhythmia.
